# Study of the Effect of Surfactants on Extraction and Determination of Polyphenolic Compounds and Antioxidant Capacity of Fruits Extracts

**DOI:** 10.1371/journal.pone.0057353

**Published:** 2013-03-05

**Authors:** Reza Hosseinzadeh, Khatereh Khorsandi, Syavash Hemmaty

**Affiliations:** 1 Food and Chemical Analysis Research Lab, Academic Center for Education, Culture and Research, Urmia Branch, University of Urmia, Urmia, Iran; 2 Institute of Biochemistry and Biophysics, University of Tehran, Tehran, Iran; University of Sassari, Italy

## Abstract

Micelle/water mixed solutions of different surface active agents were studied for their effectiveness in the extraction of polyphenolic compounds from various varieties of apples from west Azerbaijan province in Iran. The total content of polyphenolic compound in fruit extracts were determined using ferrous tartrate and Folin–Ciocalteu assays methods and chromatographic methods and compared with theme. High performance liquid chromatography is one of the most common and important methods in biochemical compound identification. The effect of pH, ionic strength, surfactant type, surfactant concentration, extraction time and common organic solvent in the apple polyphenolics extractions was studied using HPLC-DAD. Mixtures of surfactants, water and methanol at various ratios were examined and micellar-water solutions of Brij surfactant showed the highest polyphenol extraction efficiency. Optimum conditions for the extraction of polyphenolic compounds from apple occurred at 7 mM Brij35, pH 3. Effect of ionic strength on extraction was determined and 2% (W/V) potassium Chloride was determined to be the optimum salt concentration. The procedure worked well with an ultrasound bath. Total antioxidant capacity also was determined in this study. The method can be safely scaled up for pharmaceutical applications.

## Introduction

Antioxidants are reducing agents that may protect living cells from the damage caused by unstable molecules known as free radicals and reactive oxygen species (ROS) [1]. Antioxidants neutralize free radicals as the natural by-product of normal cell processes. Free radicals are molecules with incomplete electron shells in structure. Free radicals can produce from oxidation reactions [2]–[4]. Exposure to various environmental factors, including tobacco smoke and radiation, can also lead to free radical formation. These radicals can start chain reactions. The reactive oxygen species produced in cells include hydrogen peroxide, hypochlorous acid, and free radicals such as the hydroxyl radical and the superoxide anion. The hydroxyl radical is particularly unstable and will react rapidly and non-specifically with most biological molecules due to chain reactions induced by radicals [5], [6]. This species is produced from hydrogen peroxide in metal-catalyzed redox reactions such as the Fenton reaction and increase reactive radical population in the living cells. These oxidants can damage cells by starting chemical chain reactions such as lipid peroxidation, or by oxidizing DNA or proteins. Damage to DNA can cause mutations and possibly cancer, if not reversed by DNA repair mechanisms, while damage to proteins causes enzyme inhibition, denaturation and protein degradation. When the chain reaction occurs in a cell, it can cause damage or death to the cell [7], [8]. Antioxidants terminate these chain reactions by removing free radical intermediates, and inhibit other oxidation reactions. They do this by being oxidized themselves, so antioxidants are often reducing agents. Plants and animals maintain complex systems of multiple types of antioxidants, such as glutathione, vitamin C, vitamin A, vitamin E, and polyphenolic compounds (as secondary metabolites in plants) as well as enzymes such as catalase, superoxide dismutase and various peroxidases. Insufficient levels of antioxidants, or inhibition of the antioxidant enzymes, cause oxidative stress and may damage or kill cells. As oxidative stress appears to be an important part of many human diseases, the use of antioxidants in pharmacology is intensively studied, particularly as treatments for stroke and neurodegenerative diseases. Moreover, oxidative stress is both the cause and the consequence of disease. Antioxidants are widely used in dietary supplements and have been investigated for the prevention of diseases such as cancer, coronary heart disease. Antioxidants are classified into two broad divisions, depending on whether they are soluble in water (hydrophilic) or in lipids (hydrophobic). In general, water-soluble antioxidants react with oxidants in the cell cytosol and the blood plasma, while lipid-soluble antioxidants protect cell membranes from lipid peroxidation. These compounds may be synthesized in the body or obtained from the diet. As mentioned above plant products have great potential of antioxidant activity due to contain of secondary antioxidant polyphenolic compounds. Extraction of these compounds from plant and fruit materials makes great interest in pharmaceutical researches and applications of plant polyphenols [9], [10].

Phenolic compounds represent a large group of chemical substances with a variety of functions in plant growth, development, and defense. Phenolic compounds include signaling molecules, pigments and flavors that can attract or repel, as well as compounds that can protect the plant against insects, fungi, bacteria, and viruses.

There is a huge body of evidence that phenolic compounds have effects on human health and are one of the most diverse and widespread groups of natural constituents, universally distributed among vascular plants. Plant polyphenols present in the human diet are of great interest as they possess potential anti-carcinogenic properties and antioxidant activity, in their function as free radical scavengers. Also they exhibit other effects such as reducing blood pressure, and lowering incidences of cancer and cardiovascular diseases. A concern of the widespread use of phenolic compounds is the estrogenic activity these compounds may display, which impacts the hormone balance and may result in breast cancer in women. Moreover, phenolic compounds have an important role in the nutritional, organoleptic and commercial properties of agricultural foodstuffs, since they contribute sensory properties such as color, astringency, bitterness and flavor [11], [12]. Fruits and vegetables are excellent sources of phenolics. The general perception that apples are good for health has encouraged many researchers to search for the “magic” ingredient in apple. Recent epidemiological studies have shown an inverse correlation between the consumption of apple and/or related products, and many chronic diseases of humans. Most noticeably, apple consumption has been associated with lowered risk of cardiovascular disease, lung dysfunctions, and various cancers, particularly prostate, liver, colon, and lung cancers [13]. The content of phenolics in apples is affected by variety, maturity, harvesting season, processing, cultural conditions, crop load, development of infection, fruit position within the canopy, and geographic origin [14].

The methodology used to analyze phenolic compounds in apples generally includes extractions with solvents, such as methanol, ethanol, acetone, or mixtures of these with water; cleanup and further fractionation by liquid–liquid extraction (usually with ethyl acetate); and column chromatography or solid-phase extraction [15]–[19]. Finally, after the extract is concentrated, polyphenols are separated by reversed-phase high-performance liquid chromatography (RP-HPLC). Sample extraction procedures are often regarded as bottlenecks in analytical methods. Moreover, classical sample preparation techniques are both time and solvent consuming. In addition, this step accounts for at least one-third of the error generated by the analytical method. The importance of sample preparation in analytical chemistry cannot be overemphasized.

Surfactants belong to the class of compounds known as amphiphiles, molecules having both a hydrophobic and hydrophilic component [20]. The hydrophobic component is generally referred to as the tail group and hydrophilic group is known as the head. The term surfactant comes from a contraction of “surface active agent” and is defined as a material which when present at low concentrations, adsorb onto the interface, or surface, of the system and thereby alters the interfacial free energies of the interface [21]. The concept of micelles in solution was developed by James William McBain and coworkers at the University of Bristol in Bristol, England in the early twentieth century. Micelles are aggregates formed by surfactants above their critical micelle concentration (CMC) and are composed of a hydrophilic surface and hydrophobic core. This specific structure makes the micelles capable of establishing chemical and physical interactions with either hydrophilic or lipophilic substances, which can be exploited in pharmaceutical analysis [22]–[24]. These properties of surfactants make them useful in separation sciences and chromatographic applications such as drug and dye solubilization, and in analysis by micellar liquid chromatography, electrophoresis and electrochemical techniques. Also they are useful in the studying of the thermodynamic properties and stability of macromolecules such as proteins and enzymes [25]–[29]. Due to the fact that polyphenolics are the series of compound that have different chemical structures with a large range of polarity, we need suitable solutions for their extraction and identification. The properties of surfactants suggest that they may improve the extraction and separation of phenolic compounds from natural materials. If low concentrations of surfactants can improve extraction efficiency, it might be possible to safely scale up this process for pharmaceutical applications. We can use bio-surfactant in extraction of these pharmaceutical compounds in scale up conditions for healthy drug solution production from fruits and pharmaceutical herbals, safely.

In this paper, for the first time, we report on the application of surfactants in separation, extraction and determination of polyphenols from apple fruit. The effects of various experimental parameters were investigated and optimum conditions were determined.

## Experimental Methods

### Reagents and Standards

Ethanol, methanol (HPLC grade), sodium acetate, acetonitrile, glacial acetic acid, sodium potassium tartarate, di-sodium hydrogen phosphate, sodium di-hydrogen phosphate and phosphoric acid were purchased from E. Merck chemical Co. Cerium Sulfate Di-Hydrate was purchased from AppliChem chemical Co. Catechin, Epi-Catechin, Floridizine, chlorogenic acid, galic acid, quercetin, comaric acid, Rotin, Arbutin and surfactants supplied by Sigma and Fluka Companies. Botilated Hydroxy toluene (BHT) was purchased from Sigma.

### Apparatus and Instruments

All pH measurements were made at 25°C, using Metrohm 744 pH meter (Metrohm, Switzerland). Absorption spectra were recorded on a Perkin-Elmer Lambda 25, double-beam UV–Vis spectrophotometer with 1.0 cm matched quartz cells and thermostat cell holder for adjusting the temperature. An 1100 series Agilent HPLC apparatus (Agilent technologies, USA) equipped with quaternary pump, degasser and diode array detector was used. Separations carried out on a C_18_- ODS column (250×4.6 mm I.D., 5 µm particle size). Ultrasound devise (Vikenza-Italia) and Centrifuge (up to 14000 rpm, Hettich, Germany) were used.

### Samples

Apples from various varieties were collected from research gardens of Urmia University and Urmia branch of ACECR (Academic Center for Education, Culture and Research). The samples were carefully peeled and frozen. The frozen samples were milled carefully and stored in freezer until analysis.

### Sample Preparation

The ground samples were then transferred to a beaker with appropriate concentrations of surfactant aqueous solution. To all of the extraction vials 2 ml of methanolic solution of BHT (0.8%) was added for prevent of polyphenols oxidation at extraction conditions. The mixture was well mixed and homogenized using an ultrasound bath at optimum extraction time. The extracts were centrifuged at 5000 rpm for 5 minutes at 0°C. The final extracts were stored at −20°C before being analyzed.

### HPLC Conditions

An HPLC system (Agilent Technology 1100 series) equipped with a quaternary pump, an inline degasser, and a diode array detector (DAD) was used for the identification and quantification of various phenolic compounds in the samples. The binary mobile phase consisted of a 0.01% phosphoric acid buffer solution (solvent A, pH 3.6, v/v) and methanol (solvent B), and the gradient program was organized as Table 1.

**Table 1:** The gradient program of HPLC mobile phase in profiling of phenolics compounds.

Flow (mL/min)	B%	Time (min)
1	5	0
1	50	10
1	100	25

The detector was set at 280, 320, 360, and 520 nm for simultaneous monitoring of the different groups of phenolic compounds.

## Results and Discussion

### The Effect of Various Extracting Solvents

**Figure 1:**
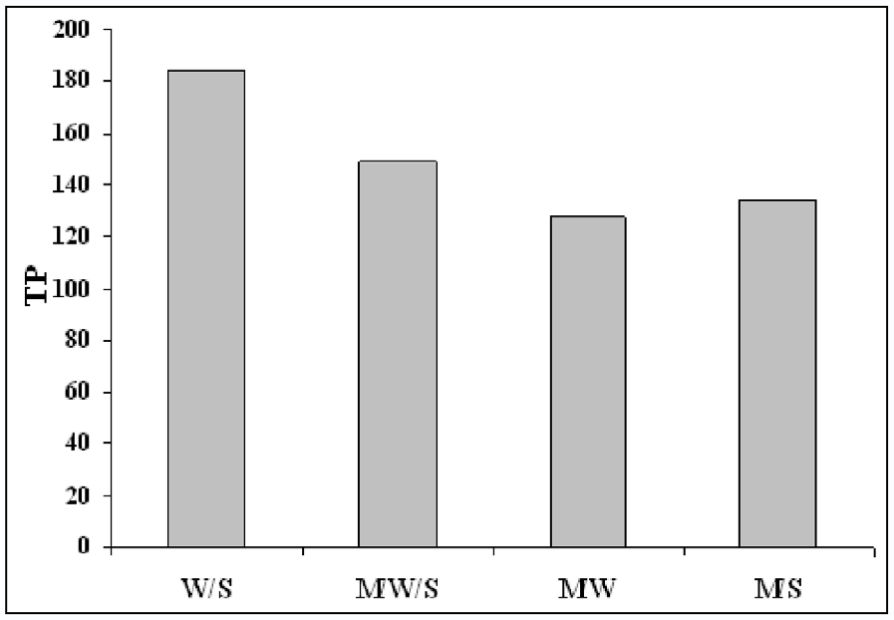
Selection of optimum extracting solution of polyphenols.

Due to the extensive range of structural properties of various polyphenolic compounds, extraction of these compounds has been a challenge for research scientists. Various solvent extraction methods have been used for phenolic compounds [30]–[39]. In all of these methods extraction was done using toxic and expensive organic solvents which have side effect for health and the environment. In addition, extraction processes are complicated and time-consuming; in this paper, the extraction of phenolic compounds with common solvents and surfactant solutions was examined. Four types of mixed solvents were considered for this study: methanol-water (80∶20) (M/W), surfactant methanolic solution (M/S), surfactant-methanol-water solution (M/W/S), and surfactant water solution (W/S). Total phenolics (TP) extracted using these mixed solvents are shown in Figure 1.

It can be seen that the surfactant aqueous solution (W/S), has the highest extraction efficiency, compared to the other extracting solutions.

Due to the fact that surfactants are amphiphilic compounds and have the ability to producing various micellar phases in aqueous and organic solutions, make them marvelous compound in extraction of various compounds. According to these abilities they can solved various hydrophilic and hydrophobic compounds in themselves micellar phases. Phenolics compounds consist of various hydrophobic and hydrophilic compositions. Extraction methods in the bases of organic solvents cannot make beneficial extraction efficiency for all of these compounds.

Thus far, no specific or appropriate extraction solvent is recommended for optimal recovery of total phenolic content from fresh sample matrix owing to the diverse chemical structures of polyphenolics ranging from simple and free to conjugated and polymerized forms (lipophilic) that might consequently affect their solubility behavior.

Also these solvents are toxic and extraction method bases of organic solvents need high amount of solvent while these solvents are harmful for human health. Surfactants micellar water solution can extract these compounds with high efficiency of extraction.

### The Effect of Surfactant Type and Concentration

**Figure 2:**
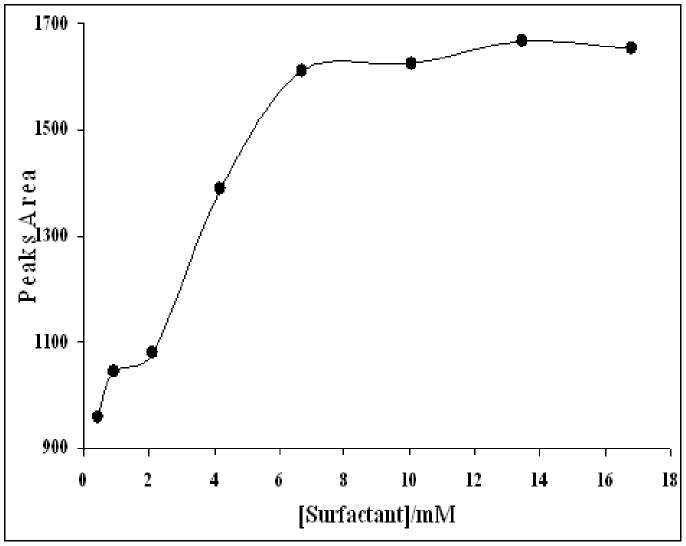
Total extracted phenolic compounds versus surfactant concentration.

For examination of surfactant type, Cethylthrimethyl ammonium bromide (CTAB), Sodium dodecyle sulfate (SDS), Triton X-100, PEG 2000, Brij 35 surfactants were selected for this study. Solutions containing CTAB produced a sediment phase and were not suitable for extraction. Non-ionic surfactants, in comparison, have a better extraction efficiency than do ionic types. Brij 35 surfactant solution has the greatest extraction efficiency of the non-ionic surfactants tested. The Effect of surfactant concentration was evaluated over a range that included concentrations higher than the critical micellar concentration of surfactant. It must be mentioned that Buthylated Hydroxy Toluene (BHT) was added to all solution because of prevention of phenolics compound from oxidation stresses. The effect of surfactant concentration on phenolics extraction is shown in Figure 2. The total concentrations of phenolics were determined using chromatograms of sample solutions.

Increasing surfactant concentration has a positive effect on extraction until 7 mM. At higher concentrations of surfactant the extraction efficiency remains constant. 7 mM of Brij-35 was considered the optimum concentration and was used in all subsequent experiments. The effect of surfactant structure in extraction of polyphenolics is due to the balance of hydrophibic and hydrophilic forces in extracting of these compounds because of the divers range of various inter molecular interactions role in extraction phenomena. Ionic surfactants make ion-pair with ionic phenolics and induced turbidity in solutions also the extraction percentage for theme are lower than non ionic surfactant.

### Effect of Ionic Strength

**Figure 3:**
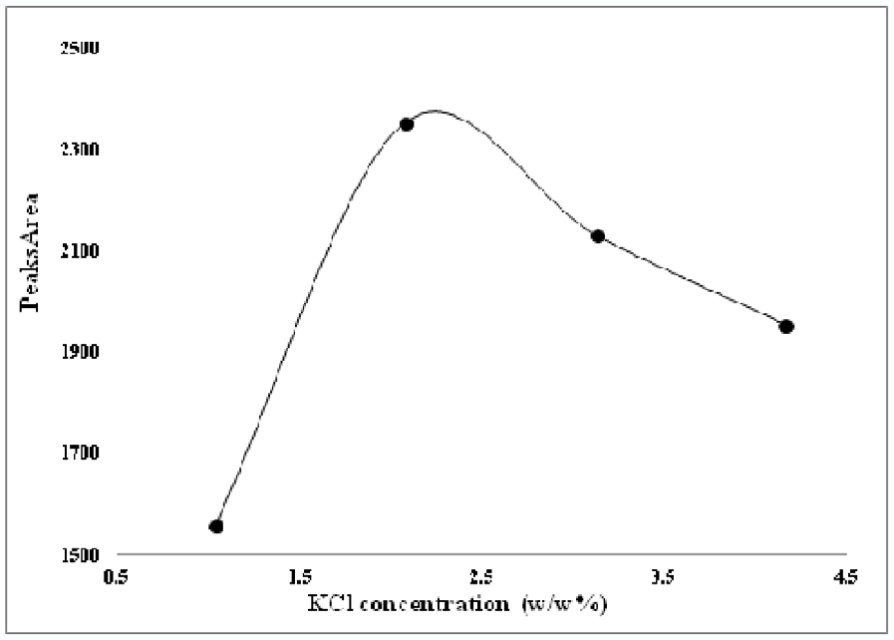
Variation of total phenolic compounds versus KCl salt concentration for selection of optimum ionic strength.

Another important factor is the ionic strength of the extracting solution. Stokes solution, 25% (w/v) potassium chloride prepared in water, was added to extracting solutions to provide specific concentration and ionic strength. Increasing the ionic strength of the extracting solution has a dual effect on the extraction process by both reducing the CMC concentration and increasing its electrostatic properties. Increasing the salt concentration resulted in the extraction of hydrophobic material in the micelle cores but blocked extraction of hydrophilic compounds, due to the electrostatic interaction and distribution. Also the salt decreased CMC of surfactant and consequently this phenomena decrease the free surfactant molecules in the stationary phase that make improve in efficiency of separation and distribution of polyphenolics compounds. As can be seen in Figure 3, 2% (w/v) KCl was selected as the optimum salt concentration for further studies.

### Effect of pH

**Figure 4:**
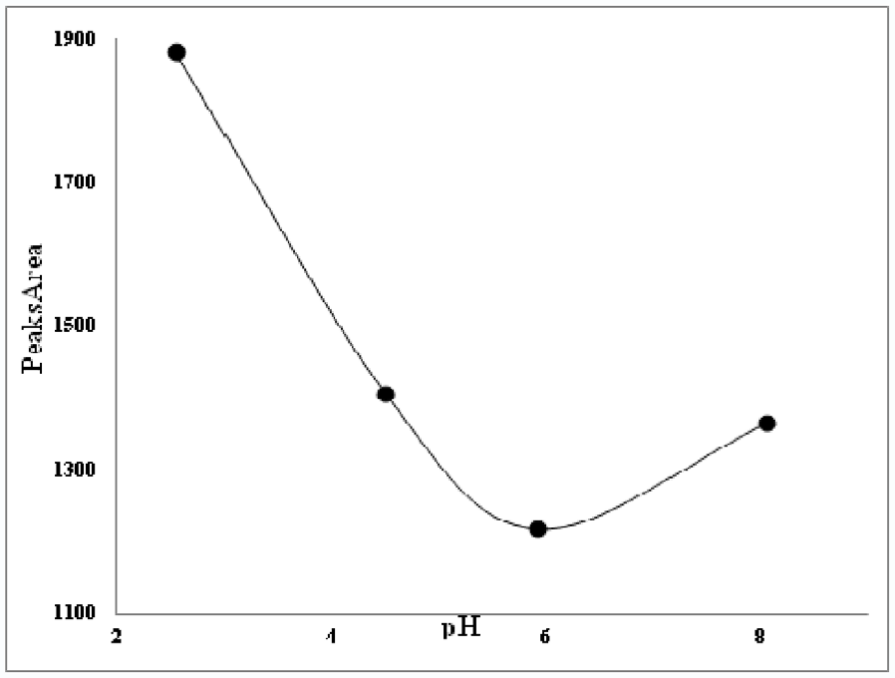
Variation of total phenolic compounds versus extracting solution pH for selection of optimum pH.

Another important parameter affecting the extraction of phenolics is the solution pH. Most of the polyphenolic compounds are in acidic form and depending on the solution pH exist in neutral or ionic forms. As can be seen in the Figure 4, when the pH value is adjusted in lower ranges, extraction of phenolic compound increases. This is due to the formation of neutral phenolic compounds and the extraction of these compounds into the micellar phase. Also hydrophilic compounds are easily dissolved and extracted in aqueous micellar solution.

### Extraction and Comparison of Phenolics Content and Antioxidant Activity in Some Apple Varieties Using Proposed Method

Total phenolic content and antioxidant activity of some apple varieties in West Azerbaijan province were measured using a new method. Total phenols were determined using the HPLC-DAD method and antioxidant assay activity were determined by methods that reported by Ozyurt [40]. The results were summarized in table 2 and 3.

**Table 2:** The phenolic compound profile and total content in various variety of apple fruit in optimum extraction conditions.

	GalicAcid	Catechin	Epi-catechin	ChlorogenicAcid	CumaricAcid	Fluoridizin	Quersetin	TotalPhenolics
**Delbar Stival**	1.19	5.01	79.83	74.49	1.357	107.21	6.84	275.927
**Red** **Delicious**	2.89	2.70	216.60	127.13	1.185	148.83	3.67	503.005
**Grani Smeet**	1.31	4.92	100.97	74.87	0.812	66.04	4.15	253.072
**Ingread meri**	1.15	1.79	158.20	141.15	0.817	90.80	1.05	394.957
**Ida Red**	2.89	1.12	36.31	143.59	0.220	46.78	8.39	239.300
**Fuji**	3.21	3.68	88.11	279.87	0.669	194.88	13.09	583.509
**Recovery%**	98.32±5.44	99.59±3.36	102.73±6.18	99.18±3.86	97.29±4.57	104.29±4.84	99.65±5.19	

**Table 3:** Antioxidant activity assay results for extracted solution of various varieties of apple fruit in optimum extraction conditions.

	Antioxidant activity (×10^−6^ M)
**Fuji**	82.4
**Red Delicious**	55.4
**Grani Smeet**	9.4
**Ingread meri**	12.4
**Ida Red**	75.3
**Delbar Stival**	33.4

In view of increasing environmental concerns about the use of organic solvents in the extraction of natural products, there has been a growing interest in “green” separation technology. Various new proposed methods are mentioned in research article dealing with phenolics compounds extraction. Nowadays applications of new methods without organic toxic and expensive solvents have great potential in various studies [41]. Using of microwave, ultrasound assisted solvent extraction have similar problems because of solvent is necessary for extraction [42], [43]. Super critical fluid extraction based on water or CO2 are reported in articles [44], [45]. According to the techniques and instrumental requirements or extraction conditions in high pressure or temperature is needed for the extraction. Our new proposed method based on bio-compatible surfactants using low amounts and concentrations of surfactant water solution with high efficiency in extraction of various polyphenolic compounds with divers’ structures and simple and inexpensive procedure, make new window to pilot plant extraction of polyphenolic compounds from plant sources as pharmaceutical natural drugs.

As mentioned in previous sections, one of the micelles properties is their ability to solubilize compounds, which are insoluble or only sparingly soluble in water; this property is of primary importance for development of analytical methods.

Micelles are dynamic microaggregates, which are approximately spherical at surfactant concentration close to the CMC; this shape is geometrically constrained. The increasing of added salt concentration in solution results in micellar growth and a change of shape from spherical to ellipsoidal. Surfactant self-organization is driven by minimization, but not complete elimination of the hydrocarbon/water contact and some methylene groups come into contact with water during a certain fraction of time [46].

Reverse phase high-performance liquid chromatography (RP-HPLC) involves a non-polar stationary phase, often a hydrocarbon chain, and a polar mobile or liquid phase. The mobile phase generally consists of an aqueous portion with an organic addition, such as methanol or acetonitrile. When a solution of analytes is injected into the system, the components begin to partition out of the mobile phase and interact with the stationary phase. Each component interacts with the stationary phase in a different manner depending upon its polarity and hydrophobicity. In reverse phase HPLC, the solute with the greatest polarity will interact less with the stationary phase and spend more time in the mobile phase. As the polarity of the components decreases, the time spent in the column increases. Thus, a separation of components is achieved based on polarity. The addition of micelles to the mobile phase introduces a third phase into which the solutes may partition. Also, the growing awareness and application of so called “green” techniques with less environmental impact may raise the profile of micellar liquid chromatography (MLC) to those who might otherwise choose a more traditional reversed phase separation. An ongoing worldwide shortage of acetonitrile also has chromatographers looking for alternative solvents, or ways to reduce organic solvent usage. The much lower concentrations of organic solvents in MLC provides an attractive alternative to RP-HPLC in terms of cost, environmental impact, toxicity, and availability of necessary materials. One of the main drawbacks of the technique is the reduced efficiency that is caused by the micelles. Despite the sometimes poor efficiency, MLC is a better choice than ion exchange liquid chromatography for separation of charged molecules and mixtures of charged and neutral species.

Several advantages of micellar eluents in comparison with classical aqueous-organic eluents can be listed as below: 1) the possibility of simultaneous separation of charged and uncharged solutes, 2) direct injection of physiological fluids due to the capability of some micellar solutions to solubilize the protein matrix of samples, 3) compatibility of mobile phases with salts and water-insoluble compounds, 4) unique separation selectivity that is due to microheterogeneity of micellar eluents and dynamic modification of stationary phase, 5) robustness of results that is caused by stabilization of surfactant monomer concentration in the presence of micelles, 6) rapid gradient capability and shorter equilibration times, 7) enhanced luminescent detection that is due to the solubilization of solutes, 8) low cost of micellar eluents, 9) safety versus expensive and flammable solvents of chromatographic grade [47].

By reducing the adsorbed surfactant on stationary phase through addition of small amounts of organic additives such as the combination of 1-butanol and triethylamine or with increasing ionic strength (decreases CMC of surfactant and reduces mono surfactant molecules adsorption on stationary phase) MLC can have efficiencies on par with RP-HPLC [47], [48]. It can be seen that addition of salt concentration makes advantages in this view of point for separation and determination of phenolic compounds in here. Also according to this fact that phenolic compounds contains extended material by various structural properties so using of surfactant make this possibility we can separate these compounds on the column in short run time by good selectivity and efficiency.

### Conclusion

This work focused on the ability of surfactants to be used in extraction and analysis of fruits phenolic compounds and in measurement of antioxidant activity. The Effects of various parameters such as pH, ionic strength, and surfactant concentration and type in the extraction of polyphenolics was examined. Obtained results shows that Brij/water solutions had the highest extraction efficiency with the following optimum conditions -; pH = 3, 7 mM surfactant concentration, 2% (w/v) KCl salt solution, using an ultrasound bath assisted extraction procedure. Total phenolic content and antioxidant activity were measured and compared with a commonly used procedure. The proposed method results in an increase in extraction efficiency. Unfortunately, anthocyanins cannot be analyzed in the presence of surfactants because of turbidity effects.

## References

[1] Sies H (1997) Oxidative stress: Oxidants and antioxidants. Experimental physiology 82 (2): 291–5. 10.1113/expphysiol.1997.sp004024 9129943

[2] German JB (1999) Food processing and lipid oxidation. Advances in Experimental Medicine and Biology 459: 23–50. 10335367 10.1007/978-1-4615-4853-9_3

[3] Davies KJ (1995) Oxidative stress: The paradox of aerobic life. Biochemical Society Symposia 61: 1–31. 10.1042/bss0610001 8660387

[4] Vertuani S, Angusti A, Manfredini S (2004) The Antioxidants and Pro-Antioxidants Network: An Overview. Current Pharmaceutical Design 10 (14): 1677–94. 10.2174/1381612043384655 15134565

[5] Rhee SG (2006) CELL SIGNALING: H2O2, a Necessary Evil for Cell Signaling. Science 312 (5782): 1882–3. 10.1126/science.1130481 16809515

[6] Valko M, Leibfritz D, Moncol J, Cronin M, Mazur M, Telser J (2007) Free radicals and antioxidants in normal physiological functions and human disease. The International Journal of Biochemistry & Cell Biology 39 (1): 44–84. 10.1016/j.biocel.2006.07.001 16978905

[7] Valko M, Izakovic M, Mazur M, Rhodes CJ, Telser J (2004) Role of oxygen radicals in DNA damage and cancer incidence. Molecular and Cellular Biochemistry 266 (1–2): 37–56. 10.1023/b:mcbi.0000049134.69131.89 15646026

[8] Stadtman E (1992) Protein oxidation and aging. Science 257 (5074): 1220–4. 10.1126/science.1355616 1355616

[9] Cao D, Li H, Yi J, Zhang J, Che H, et al (2011) Antioxidant Properties of the Mung Bean Flavonoids on Alleviating Heat Stress. PLoS ONE 6(6): e21071. 21695166 10.1371/journal.pone.0021071 PMC3112222

[10] Alvarez-Suarez JM, Dekanski D, Ristic S, Radonjic NV, Petronijevic ND, et al (2011) Strawberry Polyphenols Attenuate Ethanol-Induced Gastric Lesions in Rats by Activation of Antioxidant Enzymes and Attenuation of MDA Increase. PLoS ONE 6(10): e25878. 22016781 10.1371/journal.pone.0025878 PMC3189224

[11] Vermerris W, Nicholson R (2006) Phenolic compound biochemistry, Springer.

[12] Alonso-Salces RM, Korta E, Barranco A, Berrueta LA, Gallo B, Vicente F (2001) Pressurized liquid extraction for the determination of polyphenols in apple. J. Chromatogr. A 933: 37–43. 10.1016/s0021-9673(01)01212-2 11758745

[13] Tsao R, Yang R, Young J Ch, Zhu H (2003) Polyphenolic Profiles in Eight Apple Cultivars Using High-Performance Liquid Chromatography (HPLC). J. Agric. Food Chem. 51: 6347–6353. 10.1021/jf0346298 14518966

[14] Shahidi F, Naczk M (2004) Phenolics in Food and Nutraceuticals. CRC PRESS Boca Raton London New York Washington, D.C.

[15] Guyot S, Marnet N, Laraba D, Sanoner P, Drilleau JF (1998) Reversed-Phase HPLC following Thiolysis for Quantitative Estimation and Characterization of the Four Main Classes of Phenolic Compounds in Different Tissue Zones of a French Cider Apple Variety (Malus domestica Var. Kermerrien). J. Agric. Food Chem. 46: 1698–1705.

[16] Guyot S, Doco T, Souquet JM, Moutounet M, Drilleau JF (1997) Characterization of highly polymerized procyanidins in cider apple (Malus sylvestris var. kermerrien) skin and pulp. Phytochemistry 44: 351–357.

[17] Perez Ilzarbe J, Hernandez T, Estrella I, Vendrell M (1997) Cold storage of apples (cv. Granny Smith) and changes in phenolic compounds Lebensm. Z. Unters. Forsch 204: 52–55.

[18] Escarpa A, Gonzalez MC (1998) High-performance liquid chromatography with diode-array detection for the determination of phenolic compounds in peel and pulp from different apple varieties, J. Chromatogr. A 823: 331–337. 10.1016/s0021-9673(98)00294-5 9818410

[19] Coseteng MY, Lee CY (1987) Changes in Apple Polyphenoloxidase and Polyphenol Concentrations in Relation to Degree of Browning. J. Food Sci. 52: 985–989.

[20] Texter J (1999) Characterization of Surfactants, in Surfactants, a Practical Handbook, Lange K R, Editor, Hanser Gardner Publications, Inc.: Cincinnati.

[21] Rosen MJ (2004) Surfactants and Interfacial Phenomena, 3rd Ed., Hoboken: John Wiley & Sons, Inc.

[22] McBain JW, Cornish ECV, Bowden RC (1912) Studies of the constitution of soap in solution: sodium myristate and sodium laurate. J. Chem. Soc., Trans. 101: 2042–2056.

[23] Cudina O, Karljikovic-Rajic K, Ruvarac-Bugarcic I, Jankovic I (2005) Interaction of hydrochlorothiazide with cationic surfactant micelles of cetyltrimethylammonium bromide Colloids Surf., A. 256: 225–232.

[24] Hosseinzadeh R, Gheshlagi M (2009) Interaction and micellar solubilization of diclofenac with cetyltrimethylammonium bromide: a spectrophotometric study., Collect. Czech. Chem. Commun., 74 (3): 503–513.

[25] Berthod A, Garcia-Alvarez-Coque MC (2000) Micellar Liquid Chromatography, 1Ed. Chromatographic Science Series, Vol 83 New York: Marcel Dekker, Inc. 603.

[26] Bordbar AK, Hosseinzadeh R (2006) binding of cetylpyridinum chloride to glucose oxidase, Colloids Surf. B 53: 288–295. 10.1016/j.colsurfb.2006.09.019 17110091

[27] Bordbar AK, Hosseinzadeh R, Norozi MH (2007) interaction of a homologous series of n-alkyl trimethyl ammonium bromides with egg white lysozyme; microcalorimetric and spectroscopic study, J. Therm. Anal. Cal. 87: 453–456.

[28] Hosseinzadeh R, Sabzi RE, Ghasemlu K (2009) Effect of cetyltrimethyl ammonium bromide (CTAB) in determination of dopamine and ascorbic acid using carbon paste electrode modified with tin hexacyanoferrate, Colloids Surf. B 68: 213–217. 10.1016/j.colsurfb.2008.10.012 19084387

[29] Hosseinzadeh R, Tahmasebi R, Farhadi Kh, Moosavi-Movahedi AA, Jouyban A, et al (2011) Novel cationic surfactant ion pair based solid phase microextraction fiber for nano-level analysis of BTEX, Colloids Surf. B 84: 13–17. 10.1016/j.colsurfb.2010.11.036 21242063

[30] Antolovich M, Prenzler P, Robards K, Ryan D (2000) Sample preparation in the determination of phenolic compounds in fruits., Analyst. 125: 989–1009.

[31] Deshpande SS, Cheryan M, Salunkhe DK (1986) Tannin analysis of food products. CRC Crit. Rev. Food Sci. Nutr. 24: 401–449. 10.1080/10408398609527441 3536314

[32] Hagerman AE, Zhao Y, Johnson S (1997) Methods for determination of condensed and hydrolyzable tannins, in Antinutrients and Phytochemicals in Food, Shahidi F, Ed., ACS Symposium Series 662, American Chemical Society, Washington, D C, 209–222.

[33] Jackman RL, Yada RY, Tung MA (1987) A review: separation and chemical properties of anthocyanins used for their qualitative and quantitative analysis. J. Food Biochem. 11: 279–308.

[34] Makkar HPS (1989) Protein precipitation methods for quantification of tannins: a review. J. Agric. Food Chem. 37: 1197–1202.

[35] Markham KR (1975) Isolation techniques for flavonoids, in The Flavonoids, Harborne, J B, Mabry T J, Mabry H, Eds., Chapman & Hall, London, 1.

[36] Porter LJ (1989) Tannins, in Methods in Plant Biochemistry, vol. 1, Harborne J B, Ed., Academic Press, San Diego, CA, 389–420.

[37] Scalbert A, Monties B, Janin G (1989) Tannins in woods: comparison of different estimation methods. J. Agric. Food Chem. 37: 1324–1329.

[38] Scalbert A (1992) Quantitative methods for estimation of tannins in plant tissues, in Plant Polyphenols: Synthesis, Properties, Significance. Hemingway, R W, Laks P S, Eds., Plenum Press, New York, 259–280.

[39] Tempel AS (1982) Tannin-measuring techniques: A review, J. Chem. Ecol. 8: 1289–1298. 10.1007/BF00987762 24414735

[40] Ozyurt D, Demirata B, Apak R (2007) Determination of total antioxidant capacity by a new spectrophotometric method based on Ce(IV) reducing capacity measurement. Talanta 71: 1155–1165. 19071427 10.1016/j.talanta.2006.06.015

[41] Sulaiman SF, Sajak AAB, Ooi K, Supriatno, Seow EM (2001) Effect of solvents in extracting polyphenols and antioxidants of selected raw vegetables Journal of Food Composition and Analysis. 24: 506Comp.

[42] Wua T, Yan J, Liu R, Marcone MF, Aisa HA, Tsao R (2012) Optimization of microwave-assisted extraction of phenolics from potato and its downstream waste using orthogonal array design. Food Chemistry 133: 1292–1298.

[43] Zhang G, He L, Hu M (2011) optimized ultrasonic-assisted extraction of flavonoids from Prunella vulgaris L. and evaluation of antioxidant activities in vitro. Innovative Food Science and Emerging Technologies 12: 18–25.

[44] Zarena AS, Sachindra NM, Udaya Sankar K (2012) Optimisation of ethanol modified supercritical carbon dioxide on the extract yield and antioxidant activity from Garcinia mangostana L., Food Chemistry. 130: 203–208.

[45] Cama M, Hısıl Y (2010) Pressurised water extraction of polyphenols from pomegranate peels. Food Chemistry 123: 878–885.

[46] Seoud OA (1989) Effect of organized surfactant assemblies on acid-base equilibria (Review), Adv. Colloid Interface Sci. 30: 1–30.

[47] Boichenko AP, Loginova LP, Kulikov AU (2007) Micellar liquid chromatography (Review).Part 1. Fundamentals, retention models and optimization of separation, Methods and objects of chemical analysis, 2 (2): 92–116.

[48] Khaledi MG (1997) Micelles as separation media in high-performance liquid chromatography and high-performance capillary electrophoresis: overview and perspective. Journal of Chromatography A, 780 (1): 3–40.

